# The impact of income inequality on the fertility intention: A micro perspective based on relative deprivation

**DOI:** 10.1371/journal.pone.0311991

**Published:** 2024-12-05

**Authors:** Jun Li, Tiantian Li, Wei Wang

**Affiliations:** School of Public Economics and Administration, Shanghai University of Finance and Economics, Shanghai, China; Harran University: Harran Universitesi, TÜRKIYE

## Abstract

The relative deprivation index can reflect the income inequality faced by different individuals, which is helpful to understand the relationship between income inequality and the variability of fertility intentions. But previous studies have almost focused on the macro indicators of income inequality, ignoring individual heterogeneity in income inequality. In this study, we explore the causal relationship and potential mechanisms between income inequality and fertility intentions from the perspective of relative deprivation in income. The findings are as follows: (1) An increase in income inequality boosts individuals’ fertility intentions, and the results are still robust after using the instrumental variables two-stage least squares (2SLS) model to deal with endogeneity. (2) Mechanism analysis reveals that income inequality improves individuals’ fertility intentions through the channels of “Build hopes on children”, “Allocate more time to families” and “Put less value on children’s education”. (3) Heterogeneity analysis indicates that income inequality has a more pronounced positive impact on fertility intentions of individuals with poor education, low household assets and without pension insurance. (4) Further analysis reveals that an increase in income inequality at macro level also promote individuals’ fertility intentions. Our findings hold significant policy implications for promoting a rebound in fertility rates. When developing policies to adjust income distribution, it is necessary to consider the response of individuals’ fertility decisions to income inequality. Policymakers should ensure that efforts to improve income distribution do not inadvertently reduce the willingness of individuals to have more children.

## 1. Introduction

Social inequality has been a global concern during a long period. Increasing inequality may lead to economic instability, which is an important macroeconomic risk. Individuals’ economic decisions are often closely linked to income inequality [1–4]. Indeed, with different initial endowments or social-economic background, individuals often experience varying degrees of income inequality [5], which could affect people’s economic decisions. According to the theory of relative deprivation, when individuals feel that the distribution of social resources is highly unequal, there might be large divergence in their psychological reactions and behavioral adjustments. For example, the Confucian concept of “Fear not scarcity but inequality” is very popular in China. If the distribution of social wealth is relatively equal, everyone has more opportunities to share the fruits of economic growth and may feel more confident about the future. On the contrary, an unequal society tends to increase economic insecurity, prompting individuals to make different economic decisions. This provides a new perspective for us to understand how income inequality affects fertility decisions of individuals.

According to the theory of family economics, the fertility choice is one of the most important economic decisions for couples, as raising children can contribute to family utility [6, 7]. Different levels of income inequality often result in different fertility decisions among people [8, 9]. However, a large number of previous studies have primarily focused on the macro perspective, discussing demographic changes driven by economic development or income growth [10]. Less attention has been paid to the relationship between the distribution of the fruits of development and fertility decision-making. Only a few studies analyze the impacts of income inequality on fertility rates, but the relationship between the two is inconclusive [11–14]. The limitations of previous studies are obvious: Firstly, most studies have adopted macro-level indicators to analysis the impacts of income inequality, such as Gini Index, Theil index and so on. These indicators portray the overall income inequality of people, providing only a raw picture of the income gaps. But it is difficult to capture the actual welfare of individuals. Fertility decisions are often rational choices made by families based on their own welfare, which cannot be well-explained by income inequality indicators at the macro-level. Secondly, although some studies have explored the relationship between income inequality and fertility, none have provided a reasonable or clear explanation for this relationship. Additionally, there is a lack of empirical evidence in the analysis of the underlying mechanisms.

The impacts of income inequality on fertility intention are multifaceted. First of all, the economic value of a child almost determines the needs of parents for children. Especially in developing countries like China, “raising children for old age” is a common understanding. In poor families, having more children can provide greater support for aging parents [15–17]. High income inequality may increase the economic insecurity of disadvantaged families. When individuals feel that their own careers are hopeless, they tend to put their hopes on children. This means young couples will increase the number of children to enhance the family’s future economic security. Consequently, individuals may be more willing to have children with increasing income inequality. In addition, as income inequality increases, support within the family becomes more important. The companionship and support of family members are essential for individuals to cope with external pressures. Fertility decisions are also influenced by the relationship between family members. With greater family companionship and support, family relationships tend to be more harmonious, leading to a more positive attitude towards expanding family size [18, 19]. In terms of income inequality, people from disadvantaged families may believe that no matter how hard they try, they cannot catch up with the wealth of the rich [20]. Thus, their expected returns on education investment may decline [21, 22], leading to less investment in education. According to the Quantity-Quality (Q-Q) theory, the number of children will increase.

In this paper, we explore the causal relationship and potential mechanisms between income inequality and fertility intention based on relative deprivation in income, which allows us to better understand how income inequality affects Individuals’ fertility decisions. Specifically, we use data from the China Family Panel Studies in 2018 (referred to as CFPS2018) to construct the index of relative deprivation in income to depict income inequality at the individual micro level. With the help of relative deprivation in income, we can open the black box of the impact mechanisms of income inequality on individuals’ behaviors [23]. Our study delivers interesting results. We find that a significant increase in individuals’ fertility intentions in response to rising income inequality. Mechanism analysis indicates that increasing income inequality boosts fertility intention through three main channels: “Build hopes on children”, “Allocate more time to families” and “Put less value on children’s education”. Besides, we use the IV method to mitigate potential endogeneity, and the results are still robust.

There are several contributions to the literature. Firstly, to the best of our knowledge, our study is the first one to examine the impacts of income inequality on fertility intention from the perspective of relative deprivation in income. The relative deprivation index can reflect the income inequality faced by different individuals, providing a micro perspective to study the impacts of individual income inequality. Previous literature research on the relationship between income inequality and fertility rate mainly used income inequality indexes at the macro level [11–14]. Those indicators only provide a rough depiction of the income gap between different groups. However, the relative deprivation index can describe the actual welfare status of individuals more precisely. Fertility intentions or behaviors of people are often rational decisions based on the goal of welfare maximization, so we believe that it would be more appropriate to use the relative deprivation index to analyze the impact of income inequality on fertility intention. Secondly, using the relative deprivation index helps to analyze the mechanisms behind the impact of income inequality on fertility intentions, which might not be successful with macro indicators. Our study proposes and verifies three possible mechanisms, filling a gap about mechanisms analysis on this issue. Thirdly, we broaden the research scope of the economic consequences of income inequality. The existing research on relative deprivation in income mainly discusses its impact on health [24–26]. We extend this scope to include fertility behavior, thus making a valuable contribution to this field.

From the perspective of relative deprivation in income, we offer valuable insights into how income inequality affects fertility intentions, adding depth to our understanding of the relationship between income inequality and the variability of fertility intentions. Generally, our study can not only offer a more comprehensive explanation of historical fertility changes, but also improve predictions about future demographic transitions.

## 2. Literature review

### 2.1 The influencing factors of fertility intentions

Numerous studies have delved into the influence of individuals’ fertility intentions [27]. Existing research is mainly divided into two aspects: one is economic factors, and the other is non-economic factors. Economists usually use the “cost-benefit” of fertility to analyze the determinants of individuals’ fertility intentions. Their findings indicate that household income [24, 28], employment status [29], personal education level [30], and the social security system [31, 32], are crucial factors influencing individuals’ fertility intentions. Non-economic factors are mainly focus on the perspectives of social psychology and culture. Studies point out that confidence or belief in is an important factor affecting fertility intention [33]. When individuals are pessimistic about their own career, they tend to pin their hopes on future generations, which may lead to an increased demand for children. Moreover, some studies highlight the significant influence of cultural traditions, such as son preference culture in China on fertility intention [34–37].

### 2.2 The impact of income inequality

Income inequality is divided into income inequality at macro level and individual level. On the one hand, Gini Index and Theil Index are commonly used to describe income distribution and income inequality at macro level. These traditional indicators and related research are often hard to distinguish the relative disadvantages and advantages of individual income within groups. While these indicators may provide information about the average income of the reference group, they fail to identify income disparities among individuals in that group. In other words, these indicators can only roughly reflect overall income inequality. On the other hand, the measurement indicators of income inequality at the individual level mainly include Yitazhaki index, Kakwani index, and Podder index [38–40]. These indicators allow for a comparison with others in the reference group whose income surpasses their own, thereby obtaining an individual’s relative deprivation in income [39]. Compared with traditional macro indicators, it is more specific and can reflect people’s welfare. Hence, in order to analyze the micro impact of income inequality more precisely, it is more reasonable to employ an individual-level income inequality index [24, 25]. Relative deprivation is considered as an indicator of inequality at the individual level [41], reflecting the welfare status of individuals. It is called relative deprivation in income when income is used as a measure of relative deprivation. Nowadays, relative deprivation in income is widely recognized by scholars as an important measure of income inequality [23, 42].

Literature has broadly explored the multifaceted impacts of income inequality. From the perspective of economic development, increasing income inequality not only exacerbates economic fluctuations [43, 44] but also suppresses sustainable economic growth [45]. In terms of social development, the rise in income inequality contributes to higher crime rates [46, 47] and hampers social mobility [48], thereby worsening social instability. At the household level, income inequality significantly raises household savings rates [2, 49] and drives a rapid increase in household debt [50]. Some studies have examined the impact of income inequality on individuals’ fertility behaviors, but the results remain inconclusive [11–14]. For example, Castro and Fajnzylber [13] find a negative association between income inequality and adolescent fertility in low-income countries. Bar *et al*. [12] show that increasing inequality boosts the fertility of high-income groups while reducing the fertility of low-income groups. Japaridze [14] argues that high income inequality will leads to low fertility rates. It is worth noting that these studies have used macro-level income inequality to explain the micro-level individual’s fertility motivations or behaviors. The use of macro indicators implies an underlying assumption that the degree of income inequality faced by individuals is the same, overlooking the heterogeneity of income inequality among different individuals, which may lead to less accurate research conclusions. Although some studies have used micro-level indicators to analyze the impact of income inequality [25, 51], few have focused on individuals’ fertility behaviors. These studies mainly discuss how relative income deprivation affects individuals’ physical health [25, 52], mental health [26], and overall well-being [53].

The gaps in the literature motivate our study. We innovatively evaluate the impacts of income inequality on individuals’ fertility intentions based on relative deprivation in income. In this study, we construct the Kakwani index to measure individual income inequality and empirically validate the connection between income inequality and fertility intention. Furthermore, we discuss the underlying influencing mechanisms.

## 3. Theoretical analysis and research hypotheses

We use relative deprivation in income to represent income inequality and examine its relationship with individuals’ fertility intentions. Relative deprivation reflects the income gap between individuals and their reference group [38], indicating the opportunity for individuals from different social classes to improve their social status. Great inequality could diminish people’s motivation to pursue a better life through their own efforts. Individuals in disadvantaged groups may experience a stronger sense of income deprivation and feel more depressed about their future [39, 54]. When people lack confidence in their careers, they may shift more hope and attention to the next generation.

In developing countries, the economic value of children largely determines parents’ demand for them. For example, in China, the traditional conception of “raising children for old age” is common [15]. Children serve as a “safety net” for elderly parents [16, 17], so that having more children tends to provide more financial support for parents in old age. Therefore, when income inequality is high, fewer resources are available for old age, leading people to increase their demand for children to ensure their life for the old [55]. Conversely, when income inequality is low, there are more opportunities for upward mobility and people are more confident about their future development. This may motivate people to devote more time and money to their own careers, thus reducing their reliance on and need for their children [56, 57]. We thus propose hypothesis H1.

H1: Increasing income inequality will reduce individuals’ confidence in their future development and put their hopes on children, thus increasing their fertility intentions.

The interactions between individuals and their relatives often affect family relationships [18]. In general, when individuals spend more time with their families, family relationships tend to be more harmonious and stable, which may have a positive impact on individuals’ fertility intentions [19]. Moreover, when family members spend more time together, individuals can receive greater support for raising children. Especially for women, the husband’s involvement in household activities can greatly reduce their childbearing burden, which may improve their fertility intentions [58]. On the contrary, if couples rarely communicate with each other or if family relationships are unstable, individuals may not desire to have more children [59, 60].

Nowadays, income inequality and economic uncertainty are strongly interwoven. As income inequality increases, fewer opportunities arise for some people to improve their economic condition through hard work [61, 62]. That is, some may earn very little, no matter how much effort they put in [21]. Since increasing income inequality prevents people from moving to a higher social class, poor people may lack the incentive to work hard. They may reconsider the work-life balance, for example, focusing more on their families because they have little to lose in terms of the labor market [63]. Furthermore, in contexts of higher income inequality, individuals from lower income classes tend to experience heightened levels of loneliness [26, 64, 65]. As the company of family was found to be an effective way to against loneliness, individuals may allocate more time to their families, deepening emotional connections, and thus may improve fertility intentions. Besides, returning to the family provides people with more time to have children [59, 60]. We thus propose hypothesis H2.

H2: Increasing income inequality will encourage people to shift their focus in life from work to family, which will improve fertility intention by allocating more time to families and strengthening emotional connections.

In the analysis of individuals’ fertility behaviors, economists always use the quantity-quality trade-off theory. Due to parental altruism, individuals concern the utility derived from both the number of siblings (family size) and the quality of their children (future income of children) [66, 67]. With limited budgets, families face a trade-off between the quantity and quality of children. This theory was originally used to explain the negative correlation between family income and the number of children. As household income increases, parents tend to prioritize investing in the human capital of their children (quality), leading to smaller family sizes.

The interaction between the quantity and quality of children is also evident when analyzing the impact of income inequality on individuals’ fertility intentions. Income inequality may affect individuals’ valuation of the human capital of their children, thereby change people’s demand for children. As inequality increases, individuals may find it increasingly difficult to increase their income through their own efforts [20]. Consequently, children from disadvantaged families will hardly change their socioeconomic status even if they strive to improve their human capital, leading to a decline in the expected return from education [21, 22]. As a result, higher levels of inequality are likely to make parents to value education investment less. Under the quantity-quality trade-off of children, people may increase the size of their families. Moreover, because individuals from disadvantaged classes are typically more risk-averse [28], rising income inequality may inhibit their incentive to invest in their children’s human capital, where the probability of success and the benefits are smaller and the costs of failure are relatively greater. We thus propose hypothesis H3.

H3: Increasing income inequality will make individuals put less value on education, and fertility intention will improve under the trade-off effect of quantity and quality of children.

## 4. Data and method

### 4.1 Data and sample

We use data from the 2018 China Family Panel Studies (CFPS). This survey collects information on household economic characteristics, individuals’ demographic events, fertility intention, and other details of Chinese residents [68]. All baseline family members and their future biological or adopted children who were defined by the baseline survey in 2010 are tracked every two years. There are four parts: community data, household data, adult data and child data. To support our analysis, we merge adult data with the corresponding household data.

Given the vast regional differences in China and the need for sample representativeness, this survey employs a systematic probability sampling method involving multiple stages, implicit stratification, and probability proportional to size (PPS). The sampling process occurs in three stages: selecting administrative districts/counties, then selecting administrative villages/neighborhood committees, and finally selecting households [68]. This sampling method ensures that the selected counties and samples are nationally representative. The research sample in our study involves 153 counties.

Considering that individual’s fertility behaviors are mostly completed after the age of 60, we excluded samples aged over 60. To align with the current legal age for marriage in China (women over 20 years old, men over 22 years old), as well as the childbearing age for women (15–49 years old), we selected samples of women aged 20–49 and men aged 22–60 in the year of the interviews. After eliminating samples with missing values in selected variables, 13203 individuals remain in our study.

### 4.2 Variable description and descriptive statistics

#### (1) Dependent variable

The dependent variable in this study is fertility intention. We use the “ideal number of children” to represent fertility intention, as it has been widely acknowledged as an accurate reflection of individuals’ fertility intentions [55] and is commonly used as a proxy variable in existing literature [19, 36]. We constructed this variable based on the question, “How many children do you think are in line with your ideals?”.

#### (2) Key explanatory variable

The key explanatory variable is income inequality at the individual level, which is mainly measured by relative deprivation. Relative deprivation in income reflects the income disparity between individuals and their reference groups, and is widely used in recent literature as an indicator to measure relative income [23, 25]. Several commonly used indexes include the Kakwani index [39], Yitzhaki index [38], and Podder index [40]. Among these, the Kakwani index overcomes the shortcomings of the Yitzhaki index and the Podder index and has the characteristics of dimensionless, normality and transfer invariance. Moreover, the sum of this index for each one in a group is equal to the Gini Index of that group [39]. Thus, we adopt the Kakwani index to measure relative deprivation in income based on the net income per capita of household. The construction process of this index is as follows.

To construct the Kakwani index, we first establish a reference group. Everyone is compared with others who have higher household income within the same county, allowing us to compute the relative deprivation index for each person. The specific construction process is as follows: Let *X* be a group with a sample size of *n*, and rank the household income of the reference group in ascending order. We obtain the income vector of the reference group as *RX* = (*x*_1_,*x*_2_,…,*x*_*n*_). The relative deprivation in income of individual *i* is denoted as *RD*(*x*,*x*_*i*_), which is represented by the Kakwani index as follows:

$$RD\left(x,x_{i}\right)=\frac{1}{n\mu_{x}}\sum_{j=i+1}^{n}\left(x_{j}-x_{i}\right)=\gamma_{x_{i}}^{+}\left[\left(\mu_{x_{i}}^{+}-x_{i}\right)/\mu_{x}\right]$$ (1)

In Eq (1), $\mu_{x_{i}}^{+}$ is the average income of all individuals whose household income exceeds individual *i* in group *X*; $\gamma_{x_{i}}^{+}$ is the proportion of samples whose income exceeds individual *i* in group *X*; *μ*_*x*_ is the average income of the total sample of group *X*. According to Eq (1), we calculate the Kakwani index using the household income variable.

#### (3) Control variables

We control for factors including individual and household characteristics. Additionally, fixed effects at the provincial level have been incorporated. The specific information is showed in Table 1.

**Table 1:** Descriptive statistics.

Variable	Variable definition	N	Mean	Min	Max	SD
Fertility intention	Ideal number of children	13203	1.927	0	5	0.692
Relative deprivation in income	Using the Kakwani index	13203	0.379	0	0.950	0.224
Household income	Net income per capita of household	13203	18753.016	0	846666.667	20650.028
Gender	Male = 1, Female = 0	13203	0.589	0	1	0.492
Age	Individual’s age	13203	40.04	20	60	10.32
Education	Year of schooling	13203	8.289	0	22	4.832
Marital status	Married = 1, Other = 0	13203	0.869	0	1	0.337
Relationship with spouse	Self-assessment of the relationship with spouse	13203	4.379	1	5	0.938
Employment state	Having job = 1, Others = 0	13203	0.854	0	1	0.353
Pension insurance	Have pension insurance = 1, Others = 0	13203	0.676	0	1	0.468
Medical insurance	Have medical insurance = 1, Others = 0	13203	0.922	0	1	0.269
Number of sons	Number of sons	13203	0.790	0	5	0.695
Household asset	Natural logarithm of monetary value of total household assets	13203	11.98	0	17.74	3.263
Confidence in one’s future	How much confidence individuals have in their future	13203	4.165	1	5	0.911
The importance of children	Self-evaluation of children’s importance to parents	13201	4.518	1	5	0.825
Frequency of eating with family	Number of times to have dinner with family members every week	12537	5.625	0	7	2.353
Time spend on housework	Hours of housework per day	11674	2.144	0	19	1.910
Parental concerns for children’s education	Individuals’ concern for their children’s education as assessed by the investigator	6962	3.599	1	5	0.800
Attend tutorial classes	Attend tutoring classes = 1, Others = 0	8445	0.141	0	1	0.348
Gini Index	One of the indicators used to measure income equality within a region	13203	0.411	0.243	0.714	0.073
Kakwani Index	One of the indicators used to measure income equality within a region	13203	0.153	0.058	0.444	0.054
Theil Index	One of the indicators used to measure income equality within a region	13203	0.338	0.099	1.649	0.187

**Fig 1:**
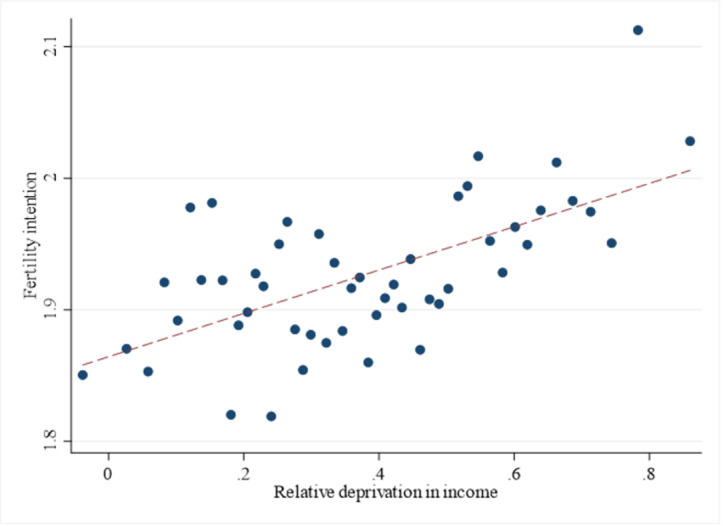
Relative deprivation in income and fertility intention.

According to Table 1, the average fertility intention of selected samples is about 1.93, indicating a relatively low demand for children among individuals in China. The mean value of relative deprivation in income is 0.379, with a standard deviation of 0.224, implying that income inequality varies greatly among individuals. The proportion of married samples stands at 86.9%. Fig 1 illustrates a scatter plot describing the relationship between relative deprivation in income and fertility intention. Notably, it shows a significant positive correlation between relative deprivation in income and fertility intention. In the following analysis, we discuss the causal relationship between relative deprivation in income and individuals’ fertility intentions.

### 4.3 Correlation analysis and VIF test

It is necessary to conduct a correlation analysis before performing the regression analysis. This is because including correlation analyses may be important to determine whether there is multicollinearity between the variables. Therefore, we conducted a correlation analysis on the core explanatory variables and control variables. The specific results are shown in Table 2. According to the results in Table 2, the correlation coefficients between all variables are less than 0.8, indicating that there is no multicollinearity problem between the variables.

**Table 2:** Correlation analysis.

	Relative deprivation in income	Gender	Age	Education	Marital status	Relationship with spouse	Employment state	Pension insurance	Medical insurance	Number of sons	Household asset
Relative deprivation in income	1										
Gender	-0.0212	1									
Age	0.0645*	0.3093*	1								
Education	-0.2534*	0.0262*	-0.1884*	1							
Marital status	0.0457*	-0.0361*	0.3225*	-0.1490*	1						
Relationship with spouse	-0.0658*	0.0792*	-0.00300	0.0547*	0.1746*	1					
Employment state	-0.1067*	0.1444*	0.0677*	0.0433*	0.0180	0.0335*	1				
Pension insurance	-0.0741*	0.0667*	0.1779*	0.0595*	0.1025*	0.0322*	0.1009*	1			
Medical insurance	-0.0199	0.0133	0.0951*	0.00240	0.1352*	0.0387*	0.0208	0.2550*	1		
Number of sons	0.1161*	0.0146	0.2725*	-0.1796*	0.3594*	0.0318*	0.0147	0.0454*	0.0624*	1	
Household asset	-0.1807*	-0.0120	-0.00280	0.1512*	0.0289*	0.0460*	0.0211	0.0573*	0.0467*	-0.0384*	1

Note: (1) * respectively represent significant levels at 1%;

(2) The correlation coefficients are Pearson correlation coefficients.

Additionally, we also examined whether there is a multicollinearity problem between independent variables through the VIF test. Table 3 presents the results of the VIF test. According to the results in Table 3, the maximum VIF value is only 1.370, which is far below 10, indicating that there is no multicollinearity problem between independent variables.

**Table 3:** VIF test.

variables	VIF	1/VIF
Age	1.370	0.728
Marital status	1.320	0.758
Number of sons	1.210	0.827
Gender	1.170	0.853
Education	1.150	0.867
Pension insurance	1.120	0.892
Relative deprivation in income	1.120	0.896
Medical insurance	1.090	0.921
Relationship with spouse	1.060	0.945
Household asset	1.050	0.949
Employment state	1.040	0.960
Mean VIF	1.150	

### 4.4 Model

Poisson Regression is suitable for analyses where the dependent variable consists of counting data, especially when the data exhibit a distinct discrete distribution. Such counting data typically comprise non-negative integers, and the maximum value is not very large, such as the number of children, the frequency of immigration and so on. Poisson regression is based on the assumption of the Poisson distribution. In this paper, the dependent variable “fertility intention” is a non-negative discrete random variable, so we adopt the Poisson model for parameter estimation. Besides, the LR test (likelihood ratio test) results from the model indicate that Prob⩾chibar2 = 1.000, indicating that the assumption of Poisson regression is satisfied.

We estimate the following regression equation:

$$Fertnum_{i}=\alpha_{0}+\alpha_{1}RD_{i}+\alpha_{2}X_{i}^{I}+\alpha_{3}X_{i}^{F}+\varepsilon_{i}$$ (2)

Where *Fertnum*_*i*_ is the fertility intention of individual *i*; *RD*_*i*_ is the relative deprivation in income of individual *i*; $X_{i}^{I}$ is a set of control variables at the individual level; $X_{i}^{F}$ is a set of control variables at the household level; *ε*_*i*_ is a random error term.

In our model, the variable *Fertnum*_*i*_ obeys the Poisson distribution with parameter *λ*_*i*_. The conditional density function of *Fertnum*_*i*_ is as follows:

$$P\left(Fertnum_{i}=n\right)=e^{-\lambda_{i}}\lambda_{i}^{n}/n!$$ (3)

Where *e* is the natural logarithm; *n* represents the value of *Fertnum*_*i*_. $\lambda_{i}^{n}=E\;\left(Fertnum_{i}|RD_{i},CV_{i}\right)$ satisfying the following condition:

$$\lambda_{i}^{n}=\exp\;\left(\alpha_{0}+\alpha_{1}RD_{i}+\alpha_{2}CV_{i}+\varepsilon_{i}\right)$$ (4)

## 5. Empirical results

### 5.1 Baseline results

To mitigate the potential influence of correlation between control variables on the regression outcomes, we introduce control variables gradually to the baseline regression. The results are presented in columns (1) to (4) of Table 4. From columns (1) to (4), the coefficients of relative deprivation in income are all significantly positive at the 1% level, suggesting that increasing income inequality has a positive impact on individuals’ fertility intentions. Additionally, the baseline regression results in column (4) indicate that: individuals with higher education or pension insurance tend to have lower fertility intention; married individuals and those who are closer to their spouses have higher fertility intentions.

**Table 4:** Baseline regressions.

	(1)	(2)	(3)	(4)
Variable name	Poisson	Poisson	Poisson	Poisson
Relative deprivation in income	0.189***	0.100***	0.101***	0.084***
	(0.049)	(0.031)	(0.032)	(0.026)
Gender		0.019***	0.019***	0.013**
		(0.006)	(0.006)	(0.006)
Age		0.000	0.000	0.002**
		(0.001)	(0.001)	(0.001)
Education		-0.008***	-0.008***	-0.005***
		(0.003)	(0.003)	(0.002)
Marital status		0.063***	0.063***	0.081***
		(0.015)	(0.015)	(0.014)
Relationship with spouse		0.013***	0.013***	0.016***
		(0.004)	(0.004)	(0.004)
Employment state		0.016	0.016	0.009
		(0.013)	(0.013)	(0.010)
Pension insurance		-0.022*	-0.022*	-0.028***
		(0.012)	(0.012)	(0.008)
Medical insurance		0.033*	0.033*	0.024
		(0.019)	(0.019)	(0.015)
Number of sons		0.120***	0.120***	0.086***
		(0.010)	(0.010)	(0.009)
Household asset			0.000	0.001
			(0.001)	(0.001)
Constant	0.583***	0.416***	0.414***	0.114***
	(0.017)	(0.053)	(0.049)	(0.041)
Provincial fixed effect	No	No	No	YES
N	13203	13203	13203	13203
Pseudo R^2^	0.0013	0.0098	0.0098	0.0173

Note: (1) ***, **, * respectively represent significant levels at 1%, 5%, and 10%.

(2) The brackets indicate the robust standard error of clustering at the county level. The following table is the same.

The results indicate that relative deprivation can significantly diminish individuals’ fertility intentions. However, it is essential to acknowledge that the empirical findings may be subject to estimation bias. Therefore, we further test the robustness of basic results.

### 5.2 Endogeneity treatment

The endogenous problems in the baseline regressions of this paper arise from two aspects. Firstly, there could be some omitted variables. Although our model includes various control variables and accounts for province-level fixed effects, certain crucial unobservable variables might still be overlooked. If omitted variables are correlated with both relative deprivation and fertility intention, it may introduce estimation bias. Secondly, there might be a problem of reverse causation. Individuals with higher fertility intention may allocate more resources and time to their children, potentially impacting their work performance and even leading to career interruptions. As a result, individuals with higher fertility intention may have lower household income, ultimately causing changes in relative deprivation.

To address the above problems, we use the IV method and choose the data on household income in 2016 as the instrumental variable because the household income in 2016 is highly correlated with its income level in 2018. Moreover, relative deprivation in income in 2018 is calculated based on income in 2018. Therefore, household income lagged by one period (2016) is negatively correlated with the relative deprivation in income of the current period [24]. However, the household income lagged by one period will not have a direct impact on current fertility intention. Thus, it satisfies the exclusivity requirements of instrumental variables. Based on the above analysis, household income in 2016, as an instrumental variable for relative deprivation in income, meets the relevant criteria for instrumental variables estimation.

We adopt the instrumental variables two-stage least squares regression (2SLS) [69] and the results are in Table 5. In column (1), the results of the first stage show that a significant negative correlation between household income in 2016 and relative deprivation in income. It indicates that the instrumental variable selected meets the correlation requirements. At the bottom of column (2), the Wald statistic is significantly greater than 10, indicating that there is no weak instrumental variable problem. In column (2), the coefficient of relative deprivation remains statistically positive at the 1% level, which is consistent with those of the benchmark regressions. Thus, we believe that endogenous problem is unlikely to be a serious threat to this study.

**Table 5:** Instrumental variable estimation.

	(1)	(2)
	First stage	Second stage
Variable name	Relative deprivation in income	Fertility intention
Household income in 2016	-0.099***	
	(0.005)	
Relative deprivation in income		0.638***
		(0.073)
Constant	1.530***	0.718***
	(0.059)	(0.127)
Control variable	YES	YES
First stage F-value		116.380
P-value		0.000
Wald statistic		2938.000
N	12363	12363

### 5.3 Robustness tests

First, the relative deprivation index essentially describes the relative income among individuals, which implies that household income may influence both individuals’ relative deprivation in income and their fertility intentions. This could lead to the observed regression results reflecting the impact of changes in household income rather than changes in relative deprivation of income. To address this, we incorporate household income as a control variable in the baseline regression, and the results are presented in column (1) of Table 6. The findings reveal that the regression coefficients of relative deprivation are essentially consistent with the baseline regressions, remaining statistically positive at the 1% level.

**Table 6:** Robustness tests.

	(1)	(2)	(3)
Variable name	Possion	Possion	Possion
Relative deprivation in income	0.086***	0.080***	0.086***
	(0.030)	(0.027)	(0.025)
Household income	0.001		
	(0.006)		
Public sector employment		-0.027**	
		(0.010)	
Constant	0.107	0.114***	0.172***
	(0.077)	(0.041)	(0.039)
Control variable	YES	YES	YES
N	13203	13203	11477

Second, the occupation of individuals plays a crucial role in determining personal income, particularly for those employed in public sectors. In this paper, the public sectors refer to government departments/party and government organs/People’s organization, public institutions and state-owned enterprises. If individuals employed in the public sector, then the dummy variable public sector employment = 1, otherwise = 0. For example, individuals employed in the public sector may have stable income sources and high social status, which may influence both their relative deprivation in income and fertility intention. To test the robustness of the basic results, we add the occupation variable into control variables. Results are presented in column (2) of Table 6, showing that the coefficients of relative deprivation remain essentially consistent with the baseline regressions.

Third, considering that fertility intentions of the unmarried individuals may be unstable, we remove unmarried samples for robustness test. In column (3) of Table 6, the coefficient of relative deprivation is still significantly positive at the 1% level.

The results of robustness test indicate that the baseline regression results are convincing.

### 5.4 Mechanism analysis

The results of baseline and robustness tests suggest that income inequality significantly increases individuals’ fertility intentions. According to the theoretical analysis above, income inequality strengthens the fertility motivation through the channels of “Build hopes on children”, “Allocate more time to families” and “Put less value on children’s education”. Next, we will analyze the mechanisms and verify the previous hypothesis.

#### (1) The channel of “Build hopes on children”

Firstly, according to the theoretical analysis, increasing income inequality will make individuals lack confidence in their own future and build more hopes on their children, which may increase their fertility intentions. Two variables are constructed to discuss this mechanism, which are “Confidence in ones’ own future” and “The importance of children”. “Confidence in ones’ own future” refers to self-evaluation of individuals’ confidence in their future. The value range is 1, 2, 3, 4, and 5; the larger the value, the higher the confidence in future. “Confidence in ones’ own future” refers to self-evaluation of individuals’ confidence in their future. The value range is 1, 2, 3, 4, and 5; the larger the value, the higher the confidence in future. Results are shown in columns (1) and (2) of Table 7.

**Table 7:** Mechanism analysis.

	(1)	(2)	(3)	(4)	(5)	(6)
	Oprobit	Oprobit	OLS	OLS	Oprobit	Probit
Variable name	Confidence in future	Importance of children	Dining with family	Time on housework	Concerns for education	Attend tutorial classes
Relative deprivation in income	-0.125**	0.129**	1.085***	0.530***	-0.348***	-0.805***
	(0.049)	(0.059)	(0.107)	(0.096)	(0.109)	(0.130)
Control variable	YES	YES	YES	YES	YES	YES
N	13203	13201	12537	11674	6962	8445

Note: The variables “Confidence in ones’ own future,” “The importance of children,” “Parental concerns for children’s education” and “Attend tutorial classes” are ordered variables. Therefore, we used the ordered probit model (Oprobit) for their estimation. The variables “Time spent on housework” and “Frequency of dining with family” are continuous variables, so we used OLS model for their estimation.

In column (1), the coefficient of relative deprivation in income is statistically negative at the level of 5%, which means that increasing income inequality will make individuals lack confidence in their own future. In column (2), the coefficient of relative deprivation in income is statistically positive at the level of 5%, which means that an increase in relative deprivation in income will enhance the important role of the child. Both results indicate that increasing income inequality boosts individuals’ fertility intentions through the channel of “place hope on children”, thus verifying Hypothesis H1.

#### (2) The channel of “Allocate more time to families”

Secondly, according to the theoretical analysis, increasing income inequality will improve fertility intentions by encouraging individuals to allocate allocating more time to families and strengthen emotional connections. Household chores are often dull, and sharing these tasks among family members may be a more positive and effective form of family companionship [58], contributing to harmonious family relationships. Therefore, we select “Time spend on housework” and “Frequency of dining with family” as proxy variables of “Allocate more time to families”. The results are in columns (3) and (4) of Table 7.

In column (3), the coefficient of relative deprivation is statistically positive at the level of 1%, which means that increasing income inequality will raise the frequency of dining with family. In column (4), the coefficient of relative deprivation in income is statistically positive at the level of 1%, which means that increasing income inequality will lead individuals to spend more time on housework. Both of the above results indicate that increasing income inequality enhances individuals’ fertility intentions through the channel of “Allocate more time to families”, thus verifying Hypothesis H2.

#### (3) The channel of “Put less value on education”

Thirdly, according to the theoretical analysis, the increase of relative income deprivation will lead individuals to put less value on education, thereby increasing their fertility intentions. We use “Parental concerns for children’s education” and “Attend tutorial classes” as proxy variables of “Put less value on education”. “Parental concerns for children’s education” refers to the attention and importance parents place on their children’s education. The value range is 1, 2, 3, 4, and 5; a higher value indicates greater concerns for children’s education. “Attend tutorial classes” refers to whether children have attended tutorial classes in the last month, yes = 1, no = 0. The results are presented in columns (5) and (6) of Table 7.

In column (5), the coefficient of relative deprivation in income is statistically negative at the level of 1%, indicating that increasing income inequality will reduce parental concerns for children’s education. Column (6) shows that the coefficient of relative deprivation in income is also statistically negative at the level of 1%, which means increasing income inequality will reduce the probability of children attending tutorial classes. These results demonstrate that increasing income inequality improve individuals’ fertility intentions through the channel of “Put less value on education”, which verify Hypothesis H3.

### 5.5 Heterogeneity analysis

We analyze the heterogeneity of the impact of increasing income inequality on fertility intention from three aspects, such as education, social security and household assets. Generally speaking, individuals with high education, pension insurance and more household wealth rely less on their children, and may also pay more attention to children’s education. Therefore, the impact of income inequality on the fertility intention of these groups might be diverse.

The results of heterogeneity analysis are showed in Table 8. According to columns (1) and (2), income inequality has a more pronounced positive impact on fertility intentions of individuals who are less well-educated. The results in columns (3) and (4) indicate that income inequality has a greater positive impact on fertility intentions of individuals without pension insurance. The results in columns (5) and (6) suggest that income inequality has a greater positive impact on the fertility intentions of individuals with low household assets.

**Table 8:** Heterogeneity analysis.

	(1)	(2)	(3)	(4)	(5)	(6)
	Possion	Possion	Possion	Possion	Possion	Possion
Variable name	Education years> = 12	Education years<12	Pension insurance = 1	Pension insurance = 0	High lnasset	Low lnasset
Relative deprivation in income	0.046	0.097***	0.081***	0.091**	0.025	0.115***
	(0.029)	(0.032)	(0.026)	(0.036)	(0.024)	(0.035)
Control variable	YES	YES	YES	YES	YES	YES
N	3938	9265	8924	4279	6599	6604

### 5.6 Further analysis

In addition, we have extended our discussion to the impact of macro-level income inequality on fertility intentions. We calculate the Gini Index, Kakwani Index and Theil Index at the county level. The information about theses indexes is shown in Table 1. In Table 9, the results show that the coefficients of Gini Index, Kakwani Index and Theil Index are all statistically positive at least at the level of 5%. This suggests that an elevation in macro-level inequality will increase individuals’ fertility intentions. Given that the micro-level relative deprivation index aggregates to form macro-level income inequality, this result serves as additional confirmation of the previously discussed findings.

**Table 9:** The impact of macro-level income inequality on fertility intention.

	(1)	(2)	(2)
Variable name	Possion	Possion	Possion
Gini Index	0.546***		
	(0.180)		
Kakwani Index		0.687***	
		(0.243)	
Theil Index			0.150**
			(0.060)
Control variable	YES	YES	YES
N	13203	13203	13203

## 6. Discussion

Understanding the relationship between income inequality and demographic transitions can help optimize future income distribution policies, especially in developing countries. Previous studies have mainly examined macro-level analyses of demographic changes linked to economic growth or income distribution. However, they have overlooked a crucial aspect: how individuals’ fertility behaviors respond to their relative income deprivation, which pertains to the micro level of income inequality. When the distribution of social resources becomes very unequal among people, there might be a large divergence in their fertility decisions as they encounter varying degrees of relative deprivation. Using data from the China Family Panel Studies in 2018, we constructed the index of relative deprivation in income and explored the causal relationship and potential mechanism between income inequality and fertility intention. This research provides an opportunity to better understand individuals’ fertility decisions, which may contribute to policy interventions and improve predictions of future demographic transitions.

We find a positive relationship between income inequality at the micro level and fertility intentions. The result challenges the conclusions of some earlier studies [13, 14], which did not consider the individual heterogeneity in income inequality and argued that income inequality leads to lower fertility rates. Previous research showed that higher income inequality generally leads to an uneven distribution of social resources, causing families to focus more on goods consumption rather than having children. However, our research provides another possibility that increasing income inequality may encourage individuals to “build hopes on children,” leading them to have more children. The results are consistent with the theoretical research findings of Takakura [70], which found that individuals at different income levels make varied fertility decisions and lower-income parents more inclined to rely on their children to improve their welfare levels. Our study also agrees with the findings of Yang *et al*. [69], which suggested that when social mobility declines, individuals are more likely to rely on children for old-age support, thereby reducing the demand for children. We emphasize the profound influence of income distribution on people’s fertility decisions, highlighting that unequal distribution of developmental dividends, not just economic growth, can alter people’s motivation to have children. When income distribution is discouraging, people may look to their children with the expectation that they will brighten the future of families.

Our study indicates that increasing income inequality can raise fertility intentions through the mechanism of “put less value on children’s education.” This finding is consistent with the theory proposed by Becker [67], which emphasizes that in the case of limited resources, there exists a trade-off and substitution relationship between the quantity and quality of children. To some extent, high income inequality tightens the budget constraints of disadvantaged families, thus may amplifying the substitution effect of the quality and quantity of children. Moreover, income inequality might also reduce the expected return on education. Poor families with limited education are less willing to invest in human capital, trapped in a vicious cycle of poverty and high fertility. This implies that “the poorer they are, the more children they will have; the more children they have, the poorer they will become.” Under the trend of declining fertility rates, governments should pay more attention to mitigating the substitution effect between the quantity and quality of children.

Furthermore, the results also indicate a possible positive effect of income inequality, as it prompts individuals to “allocate more time to families,” thereby strengthening emotional connections among family members and increasing their fertility intentions. Previous studies often emphasize the negative effects of income inequality on families and individuals [25, 26, 52, 71]. Our study identifies a positive effect of income inequality, highlighting the dual nature of the issue. If governments can guide individuals to accept the existence of equitable income deprivation and establish family support policies that encourage couples to have more children, there might be a rebound in the declining fertility rate.

## 7. Conclusion

The study indicates that an increase in income inequality does not always lead to a decline in fertility intentions. Considering the individual heterogeneity of income inequality, we find that people’s willingness to have children tends to increase as the relative deprivation index increases. Furthermore, we identify three channels through which income inequality boosts fertility intentions: “Build hopes on children”, “Allocate more time to families” and “Put less value on children’s education”.

The findings pose a couple of intriguing questions about the broader implications of income inequality on social dynamics. In a changing society, income inequality is a double-edged sword. While high income inequality is often perceived negatively due to its potential to create economic uncertainty and undermine the confidence and effort of individuals, it can also have some positive aspects. For example, people may spend more time with their family, tending to improve family relations and keep a harmonious family atmosphere, which can increase fertility intentions. Therefore, it is necessary to prevent the negative effects caused by income inequality while actively leveraging the potential positive effects it may bring.

The theoretical significance of our findings extends beyond the scope of this study. We open up new avenues for analyzing the complex relationship between income inequality and fertility decisions in the background of the accelerating changes in social structure. Policymakers should fully consider individuals’ fertility decisions when introducing policies related to income distribution. It is also critical to pay attention to individuals’ fertility motivations and to promote a fertility-friendly society.

In conclusion, our findings have sparked significant discussions about the income distribution and fertility behaviors, especially when considering individuals’ initial endowment. In situations where there is a large income gap among residents, macro-level income inequality indexes cannot distinguish between the effects of income growth and income inequality. Conversely, changes in people’s relative deprivation index in income often reveal welfare differences in among residents. This consideration of individual heterogeneity is crucial for further discussing individuals’ behavioral responses in an environment of income inequality, which helps us assess the impact of income inequality on fertility behaviors and its potential mechanisms. The study enriches academic discourse and provides new implications for predicting the influence of increasing inequality on demographic transitions.
